# Fetal liver-derived mesenchymal stromal cells augment engraftment of transplanted hepatocytes

**DOI:** 10.3109/14653249.2012.663526

**Published:** 2012-03-16

**Authors:** Meghnad Joshi, Pradeep B. Patil, Zhong He, Jan Holgersson, Michael Olausson, Suchitra Sumitran-Holgersson

**Affiliations:** 1 Department of Surgery, Port St Lucie, Florida, USA; 2 Vaccine and Gene Therapy Institute-Florida, Port St Lucie, Florida, USA; 3 Department of Clinical Chemistry and Transfusion Medicine, University of Gothenburg, Sahlgrenska University Hospital, Gothenburg, Sweden

**Keywords:** fetal liver cells, hepatocyte transplantation

## Abstract

**Background aims:**

One important problem commonly encountered after hepatocyte transplantation is the low numbers of transplanted cells found in the graft. If hepatocyte transplantation is to be a viable therapeutic approach, significant liver parenchyma repopulation is required. Mesenchymal stromal cells (MSC) produce high levels of various growth factors, cytokines and metalloproteinases, and have immunomodulatory effects. We therefore hypothesized that co-transplantation of MSC with human fetal hepatocytes (hFH) could augment *in vivo* expansion after transplantation. We investigated the ability of human fetal liver MSC (hFLMSC) to augment expansion of phenotypically and functionally well-characterized hFH.

**Methods:**

Two million hFH (passage 6) were either transplanted alone or together (1:1 ratio) with green fluorescence protein-expressing hFLMSC into the spleen of C57BL/6 nude mice with retrorsine-induced liver injury.

**Results:**

After 4 weeks, engraftment of cells was detected by fluorescence *in situ* hybridization using a human-specific DNA probe. Significantly higher numbers of cells expressing human cytokeratin (CK)8, CK18, CK19, Cysteine-rich MNNG HOS Transforming gene (c-Met), alpha-fetoprotein (AFP), human nuclear antigen, mitochondrial antigen, hepatocyte-specific antigen and albumin (ALB) were present in the livers of recipient animals co-transplanted with hFLMSC compared with those without. Furthermore, expression of human hepatocyte nuclear factor (HNF)-4α and HNF-1β, and cytochrome P450 (CYP) 3A7 mRNA was demonstrated by reverse transcriptase-polymerase chain reaction (RT-PCR) in these animals. In addition, significantly increased amounts of human ALB were detected. Importantly, hFLMSC did not transdifferentiate into hepatocytes.

**Conclusions:**

Our study reports the use of a novel strategy for enhanced liver repopulation and thereby advances this experimental procedure closer to clinical liver cell therapy.

## Introduction

Currently, the clinical use of adult hepatocyte transplantation remains limited to the treatment of metabolic liver disease or specific inborn errors of metabolism (1, 2). Most studies have failed to show beneficial effects on patient survival and have included only a limited number of patients per group (3). The suggested procedures also need repeated cell infusions and large numbers of cells for transplantation. More importantly, there are many unresolved issues, such as the use of marginal organ donors for the isolation of hepatocytes for clinical transplantation and a lack of feasible methods for increasing the proliferative capacity of adult hepatocytes and their maintenance in culture (1, 2). Several reports have demonstrated the successful engraftment of hepatocyte stem/progenitor cells in experimental models. However, the numbers of cells actually detected are less than 20%, suggesting a great loss of the total transplanted cells (4).

Human fetal hepatocytes (hFH) are in focus as attractive candidates for cell-based therapies. Human Liver Progenitor Cells (HLPC) are capable of self-renewal and terminal differentiation into multiple lineages (5). Previously, we have identified a population of hFH expressing the markers CD117 ^+^ CD34^+^ Lin^−^ that could be expanded successfully *ex vivo* more than 4000-fold over the input numbers (6). These cells could be maintained with stable morphology and phenotype for several passages. When cells in various passages were transplanted into animals with acute liver injury, they exhibited functional differentiation into hepatocytes, cholan-giocytes and sinusoidal endothelial cells (6).

If HLPC are to be used as a successful therapeutic modality, it is very important to understand the factors that allow progenitor cell integration and engraftment. Mesenchymal stromal cells (MSC), also known to be precursor cells for stromal tissue that support hematopoiesis (7), have high prolifera-tive capacity, some immunomodulatory effects (8, 9), and produce high levels of various growth factors, cytokines and metalloproteinases (10, 11). All these characteristics make this cell population a good candidate for facilitating expansion of other specialized cell types lacking these factors.

Therefore, we tested the hypothesis that co-transplantation of MSC together with fetal hepato-cytes would help augment hepatocyte cell engraftment after transplantation. We postulated that co-transplantation with MSC would provide a more suitable and advantageous microenvironment that would facilitate hFH engraftment.

## Methods

### Cell isolation from human fetal liver

Human fetal liver tissue was obtained with appropriate ethical permission from the local ethics committee at Sahlgrenska University Hospital (Gothenburg, Sweden), in accordance with Swedish guidelines. Tissues were obtained from legally aborted first trimester fetuses between 6 and 11 weeks of gestational age. A single-cell suspension was prepared with the use of a cell strainer (70 μm). The number and viability of the cells was assessed by trypan blue dye exclusion test and cell viability was more than 90% (*n* = 3). All the donors had been screened serologically for syphilis, toxoplasmosis, rubella, cytomegalovirus, parvovirus and herpes simplex virus types 1 and 2, and by Quantitative PCR (Q-PCR) detection for hepatitis B and C, and human immunodeficiency virus 1 and 2.

### Isolation of human fetal liver-derived MSC

Human fetal liver MSC (hFLMSC) were isolated using a human MSC enrichment isolation kit (Stem Cell Technologies, Vancouver, Canada) as described by the manufacturer. hFLMSC were negatively selected using a cell-depletion cocktail kit (Stem Cell Technologies), including monoclonal antibodies to lineage-specific cell-surface antigens (glycophorin A, CD3, CD14, CD19, CD66b and CD38). CD117 + CD34^+^ Lin^−^ hFH were isolated by magnetic cell sorting as described previously (6).

### Media composition for hFH and hFLMSC

Isolated hFH were centrifuged at 50 *g* for 5 min and the cell pellet was resuspended in 20 mL Dulbecco's Modified Eagle's Medium (DMEM) (Lonza ApS, Copenhagen, Denmark). The medium was supplemented with Vascular Endothelial Growth Factor (VEGF) (5 ng/mL; Invitrogen, Stockholm, Sweden), interleukin (IL)-6 (2 ng/mL; Invitrogen), Hepatocyte Growth Factor (HGF) (30 ng/mL; Invitrogen), Epidermal Growth Factor (EGF) (20 ng/mL; Millipore, Solna, Sweden), Fibroblast Epidermal Growth Factor (FGF) (10 ng/mL; Invitrogen), 5% (v/v) non-essential amino acids (NEAA), 5% (v/v) sodium pyruvate, 5% (v/v) Penicillin/Streptomycin solution (PEST) and 5% (v/v) L-glutamine (Invitrogen). Cells were cultured in collagen-coated cell culture flasks (Nunc, Roskilde, Denmark) at 37°C in a 5% CO_2_ atmosphere. The medium was changed once every 3 days and culture was split 1:2 once every fourth day.

The hFLMSC were cultured on 1% gelatin-coated flasks in MesenCult®™ medium (Stem Cell Technologies), which was further supplemented with 10% fetal bovine serum (FBS), 1% penicillin-streptomycin, and 1% L-glutamine (GIBCO, Invitrogen). The culture medium was changed every third day.

Both cell types were expanded in culture prior to use in various analysis. hFH from passages 5-6 and hFLMSC from passages 8-10 were used for characterization and transplantation.

### Production of recombinant lentivirus and transduction of MSC

The lentiviral transfer vector pHR'EFlaGFPSIN, packaging plasmid pCMVΔ8.91 and envelope plas-mid pMD. G were used to produce recombinant virion particles as described previously (12). For transduction, hFLMSC were incubated with concentrated pHR'EFlaGFPSIN virions in 25-cm^2^ flasks at a multiplicity of infection (MOI) of 10 at 37°C in a 5% CO_2_ atmosphere overnight. After virus incubation, cells were washed twice with phosphate-buffered saline (PBS) and continuously cultured for 2 days in culture medium. The cells were then frozen in liquid nitrogen for future applications.

### Phenotypic analysis of hFLMSC by flow cytometry

Phenotypic characterization of cultured hFLMSC (passages 8, 10 and 11) by flow cytometry was performed using directly conjugated primary antibodies, including CD45-fluorescein isothiocyanate (FITC), CD34-phycoerythrin (PE), CD29-PE, CD54-PE, CD29-PE, CD106-PE, CD166-PE, CD44-PE, CD73-PE and CD29-PE (all from Becton Dickinson, Stockholm, Sweden). Respective FITC and PE isotype controls were used as negative controls. Briefly, 5 × 10^5^ cells were distributed in tubes and 5 μL antibody was added directly to cells in PBS, and incubated for 30 min at 4°C. The cells were then washed with 2 mL PBS and fluorescein isothiocya-nate (FACS) analysis was performed immediately on a BD FACS Aria (Becton Dickinson, Stockholm, Sweden). Data acquisition and analysis were performed using CellQuest (Becton Dickinson).

### Phenotyping of cells using immunocytochemical analysis

Immunofluorescent staining of the cultured hFH and hFLMSC was performed on collagen-coated glass slides (BD Biosciences, Franklin Lakes, NJ, USA). Cells were cultured for 1 day in their respective media. Cells were washed twice with PBS, then fixed in ice-cold acetone:methanol (30% acetone in methanol) for 5 min. Unspecific binding was blocked with 10% goat or donkey serum. The cells were incubated overnight at 4°C with antibodies specific for human cytokeratin (CK)8 (Santa Cruz Biotechnology, Santa Cruz, CA, USA) diluted 1:100, CK18 (Santa Cruz Biotechnology) diluted 1:100, CK19 (Santa Cruz Biotechnology) diluted 1:100, hepatocyte antigen (Santa Cruz Biotechnology) diluted 1:100, Cysteine-rich MNNG HOS Transforming gene (c-Met) diluted 1:100 (RDI, Concord, MA, USA), alpha-fetoprotein (AFP; NeoMarker, Kalamazoo, MI, USA) diluted 1:100, and albumin (ALB; Bethyl Laboratories Inc., Montgomery, TX, USA) diluted 1:100. Antibodies were diluted in 1% bovine serum albumin (BSA)/PBS. After washing (2XPBS for 5 min), cells were incubated with Alexa 488-conjugated goat anti-mouse, goat anti-rabbit or donkey anti-goat IgG secondary antibodies (Invitrogen) for 40 min. After washing (4 × PBS for 5 min), cells were counterstained with 4',6-diamidino-2-phenylindole (DAPI) for 1 min. Antibody binding was detected in an IX 81 Olympus fluorescence microscope (Olympus Sverige AB, Solna, Sweden).

### Cytokine levels in hFLMSC culture supernatants determined by enzyme-linked immunosorbent assay

As MSC are known to produce high levels of various cytokines and chemokines, we analyzed the supernatants from hFLMSC cultures in passages 6, 7, 8 and 10 for these factors. Relative quantitation of inflammatory cytokines and chemokines was performed using a Multi-Analyte ELISArray Kit (SABioscences Corporation, Frederick, MD, USA). Human cytokine levels [IL-1α, IL-lβ, IL-2, IL-4, IL-6, IL-8, IL-10, IL-12, IL-17A, interferon (IFN)-γ, tumor necrosis factor (TNF)-α, and granulocyte-macrophage colony-stimulating factor (GM-CSF)] in supernatants were measured with a sandwich enzyme-linked immunosorbent assay (ELISA) according to the manufacturer's protocol.

### RNA isolation and reverse transcription-polymerase chain reaction

Total RNA was isolated from hFH, hFLMSC, primary adult hepatocytes and liver tissue from transplanted mice using a DNA/RNA/protein isolation kit (Nor-gen Biotek Corp., Thorold, Canada). The medium was aspirated, the cells washed with 2 X PBS and 600 μL cell lysis buffer with ethanol added directly to each flask. Snap-frozen liver tissue from transplanted mice was homogenized in a pestle and mortar with 600 μL cell lysis buffer. RNA was extracted according to the manufacturer's instructions. The RNA concentration was determined by ultraviolet (UV) absorbance at 260 nm using a DU 730 spectrophotometer (Beckman Coulter, Fremont, CA, USA).

One microgram of total RNA was reverse-transcribed (RT) into cDNA at 50°C using an Advantage® first-strand synthesis kit (Clontech, Mountain View, CA, USA). The cDNA samples were subjected to polymerase chain reaction (PCR) amplification using primers specific for human ALB, AFP, CK19, cytochrome P450 (CYP) 3A4, CYP3A7, hepatocyte nuclear factor (HNF)-1α, HNF-1β, HNF-4α and Glucose-6-phosphate dehydrogenase (G6PD) (Supplement M&M). The primers were selected in order to anneal to one upstream and one downstream exon with an intervening intron. Amplification conditions were as follows: initial denaturation at 95°C for 5 min was followed by cycles of denaturation at 95°C for 45 s, annealing at 50-65°C for 15 s, extension for 1 min at 72°C, and a final polymerization at 72°C for 10 min. PCR products were analyzed by gel elec-trophoresis in 2% agarose gels, stained with ethidium bromide, visualized on a The Gel Doc XR documentation system (BioRad, Sundbyberg, Sweden) and documented using Quantity One 1-D analysis software. cDNA from hFH and hFLMSC were used as controls.

### In vitro functionality testing of hFH and hFLMSC

One million hFH (passage 8, 10 and 11) and hFLMSC (passage 6, 8 and ll)/mL were used to prepare cell lysates. Cell extraction buffer (catalog number FNN0011; Invitrogen) was used along with 1 HIM phenylmethanesulfonylfluoride (PMSF) and protease inhibitor cocktail (P-2714; Sigma, Stockholm, Sweden). The protein concentration was determined using the Bradford method. The urea concentration was determined by quantitative colo-rimetric urea assay as described by Jacob *et al.* (13). The ammonia content was determined with a quantitative colorimetric assay kit (AAOIOO; Sigma). The superoxide dismutase (SOD) activity was determined with a spectrophotometer-based assay kit (OxisRe-searchTM, Foster City, CA, USA).

### Transplantation of hFH and hFLMSC in mice

The experiments were approved by the local animal ethics committee and performed in accordance with national and institutional regulations. Liver injury was induced in C57BL/6 nude mice (*n* = 44) of approximately 22-28 g body weight by intrap-eritoneal injection of retrorsine (70 mg/kg; Sigma, St Louis, MO, USA). The retrorsine working solution was prepared as described by Zhou *et al.* (14) and used immediately after preparation. Three weeks after retrorsine treatment, under general anesthesia, all C57BL/6 nude mice had a partial hepatectomy (30%). Single-cell suspensions of hFH and hFLMSC were injected (2 × 10^6^ cells in 200 μL DMEM) either alone or together (1:1) over 10-15 s into the spleen (w = 12 each). The sham group (*n* = 8) received DMEM medium alone. After securing homeostasis, the abdominal incision was closed and animals were monitored closely until recovery from anesthesia.

### Sample retrieval and processing

The mice were killed 4 weeks after transplantation. The liver was excised from each animal. Biopsies of 1-2 cm^3^ from each liver were snap-frozen in liquid nitrogen and stored at -80°C and used further for RT-PCR and immunofluorescence analyzes. The rest of the liver tissue was fixed at 4°C for 24 h in 10% buffered (pH 7.4) formalin. Five-micrometer thick cryosections were air-dried and fixed in cold 30% acetone in methanol for 10 min, and analyzed further by immunofluorescence. Formaldehyde-fixed samples were dehydrated in an alcohol series, treated with xylene, and embedded in paraffin wax. Four-micrometer sections were cut and placed on superfrost-plus microscope slides (Menzel GmbH&Co KG, Braunschweig, Germany).

### Immunohistochemical analysis of mouse liver

Liver sections were deparaffinized in xylene for 30 min. Immunohistochemistry was performed using the biotin-peroxidase complex method. Briefly, antigen retrieval was achieved by incubating the slides in 10 HIM citrate buffer, pH 6.0, in a pressure cooker at 95°C for 20 min, followed by a 20-min cooling period. Endogenous peroxidase activity was quenched with 3% H_2_O_2_ in methanol and non-specific binding was blocked with 5% horse serum. The slides were incubated overnight at 4°C with anti-human AFP diluted 1:200 in PBS, human nuclear antibody diluted 1:100 (Millipore, Stockholm, Sweden), human c-Met diluted 1:100 (RDI), human cytokeratin 8 (1:200), 18 (1:200) and 19 (1:200; Santa Cruz Biotechnology), human mitochondria diluted 1:200 (Millipore), and human hepatocyte-specific antigen (Hep Ag) diluted 1:100 (Santa Cruz Biotechnology). The slides were then washed three times with PBS/Tween-20 for 10 min. Biotinylated horse anti-rabbit or mouse secondary antibody (Immpress reagent kit; Immunkemi AB, Jarfalla, Sweden) was incubated for 40 min at room temperature. After washing (4 X PBS/Tween-20), color was developed using 3,3'-diaminobenzidine tet-rahydrochloride (DAB; Immunkemi AB). Finally, sections were stained with Gill's hematoxylin for 20 s. Stained slides were dehydrated, cleared in xylene and mounted in di-n-butyl phthalate xyline (DPX; Merk Ltd, Mumbai, India). Experiments were accompanied by negative and positive control staining to detect possible non-specific signals. Negative controls were processed by replacing the primary antibody with diluents only. Liver tissue from healthy or sham-transplanted mice was also used to detect possible non-specific signals in the staining. Sections of fetal liver, cancerous and normal adult liver tissue were used as controls for different antibodies.

The green fluorescence protein (GFP) signal was detected in unfixed liver sections. Briefly, sections were air dried, counterstained with DAPI and mounted with aqueous mounting medium (Vector Laboratories, Burlingame, CA, USA). Immunofluorescent double staining for human ALB^+^ nuclei^+^ or Ki67^+^ CK8^+^ cells was performed on the mouse liver sections. Briefly, species-specific protein blocking was done for 30 min. Goat anti-human ALB diluted 1:200 (Bethyl Laboratories Inc.), mouse anti-human nuclei diluted 1:100, Ki67 diluted 1:200 (Abeam pic, Cambridge, UK) and human cytokeratin 8 diluted 1:200 were added and incubated overnight at 4°C. The slides were then washed three times with PBS. Primary antibody was detected using either anti-goat Alexa 488/594- or anti-mouse Alexa 488/594-conjugated secondary antibodies (1:200 with PBS). Sections were counterstained with DAPI as before, mounted with aqueous mounting medium (Vector Laboratories), and examined under a fluorescence microscope (Olympus).

### Fluorescent *in situ* hybridization

To detect human cells within the mouse liver, fluorescent *in situ* hybridization (FISH) was carried out using a human-specific DNA probe. Paraffin sections of 4 μm thickness were cleared and rehydrated as before, washed in 4 × saline sodium citrate buffer (SSC) for 30 min on a shaker, and then kept overnight in 1 M NaSCN. Sections were dehydrated in serial changes of ethanol, 70%, 90% and 100%, 3 min each, and left to air-dry. Denaturation of sections was then done by applying 70% formamide in 2 × SSC, pH 7, and incubation at 80°C for 3 min. Sections were plunged into ice-cold 70% ethanol for 3 min, dehydrated in 90% followed by 100% ethanol 3 min each, and left to air-dry. Finally, the denatured pre-annealed human alpha and classical satellite probes specific to each chromosome directly labeled with red or green fluorophore (Aquarious probes, LPE R/G; Cytocell, Cambridge, UK) were added, and sections were covered, sealed and kept for overnight hybridization. The tissue samples were then mounted in Vecta shield aqueous mounting media with DAPI (Vector Laboratories). Slides were viewed with an Olympus UV lamp-equipped microscope carrying a triple-band pass filter unit (Chroma Technology, Brattleboro, VT, USA).

### Human ALB and c-Met quantification

Human ALB secretion into the serum of transplanted animals was measured with a sandwich ELISA (Bethyl Laboratory) according to the manufacturer's instructions. Human c-Met concentration in liver lysates was similarly measured in a sandwich ELISA (Invitrogen).

### Quantification of transplanted hFH and hFLMSC

To quantify the number of engrafted and proliferating cells, 200 serial sections of 5-μm thickness were sectioned from the 1-cm^3^ tissue piece and double-stained for expression of human ALB/human nuclei or Ki67/human CK8, respectively. The numbers of engrafted MSC were established by enumerating GFP + cells in the Co tansplanted hFH and hFLM-SCs (CoTx) and hFLMSCs transplanted (MTx) groups. At a 40 × magnification, the visual field of the CCD (DP72, Olympus) camera corresponds to an area of 0.06 mm^2^. Five such visual fields on each section (derived from the liver of each mouse in the different groups) centered around randomly picked portal or central regions were counted, corresponding to a surface area of 0.3 mm^2^/section. The number of engrafted cells on a 1-mm^2^ section was extrapolated from the number of cells/0.3 mm^2^ by multiplying by a factor of 3.33. This number was again multiplied by 200 to get the number of cells/mm^3^. The number of cells on each section was calculated twice, mostly by the same examiner.

### Statistical analysis

Data are presented as mean values with standard deviations. Statistical analysis of *in vitro* functionality *(n=* 12) was done using a two-tailed Student's t-test. Statistical differences in the numbers of transplanted cells was assessed with a non-parametric Mann-Whitney test (w = 4). A P-value<0.05 was considered statistically significant. Statistical analysis was performed using the SPSS 17.0 software (SPSS Inc., Chicago, IL, USA).

## Results

### hFH express all hepatocyte markers

**Figure 1:**
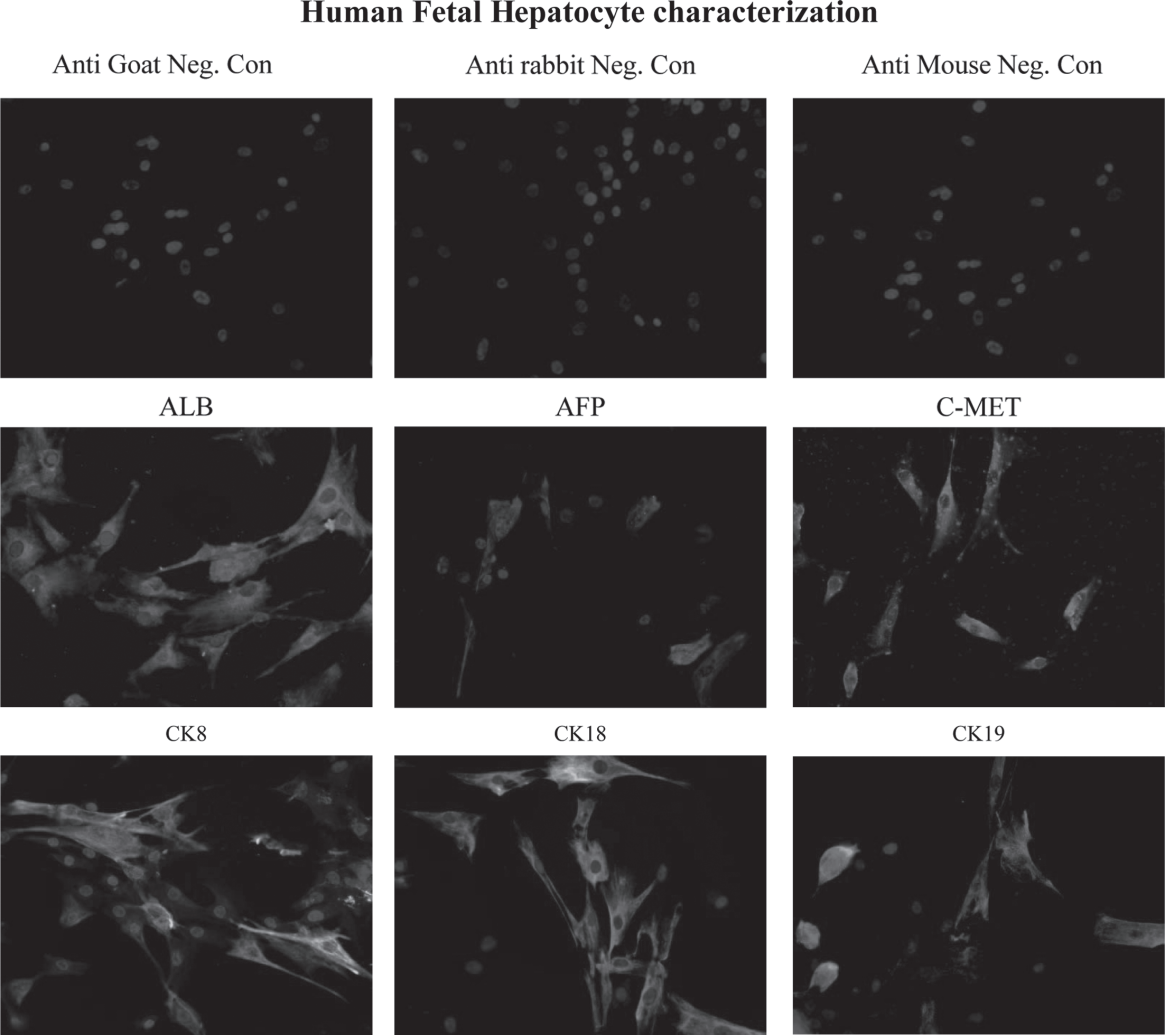
Expression of human Hep Ag in fetal hepatocytes. Fetal hepatocytes were examined for expression of various liver-specific markers, such as ALB, AFP, c-Met, CK8, CK18 and CK19, using immunocytochemistry. First row: negative control slides in which anti-goat, -rabbit and -mouse secondary antibodies were used without primary antibodies. Magnification 40 X. Second row: expression of ALB, AFP and c-Met in fetal liver cells detected (green staining) by immunofluorescence staining. Magnification 40 X.Third row: fetal hepatocyte colonies expressing CK8, CK18 and CK19 (green staining). Magnification 40 X.

The expression of ALB, AFP, CK8, CK18, CK19 and c-Met in cultured hFH was examined using immunocytochemistry (Figure 1). ALB expression was frequent and intense in hFH, while AFP expression was observed in a few cells. Cells expressing CK8, CK18, CK19 and c-Met were also found in the hFH cultures.

### hFLMSC express typical MSC markers

**Figure 2:**
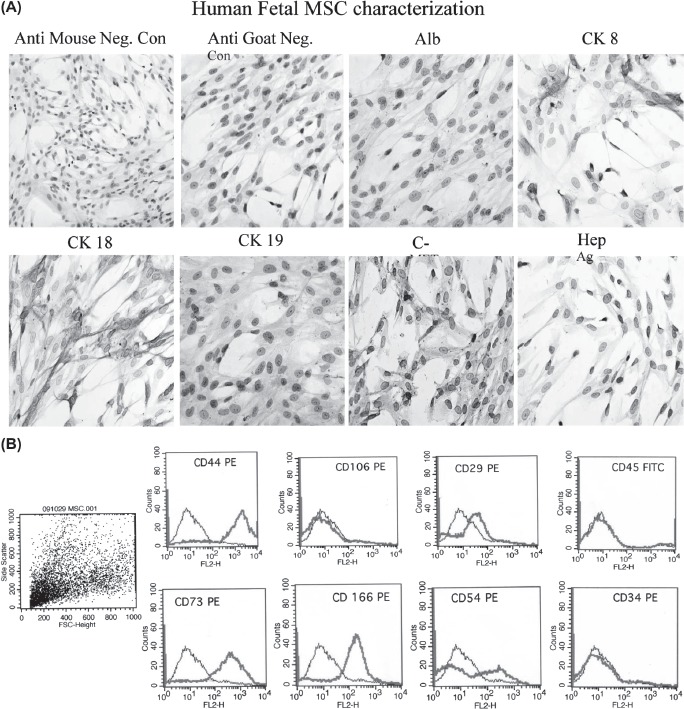
Phenotypic analysis of fetal liver MSC. (A). The expression of hepatocyte-specific markers such as ALB, CK8, CK18, CK19, c-Met and Hep Ag in hFLMSC was examined by immunocytochemistry. First row: negative controls, anti-goat and -mouse secondary antibodies. ALB staining was not detected in hFLMSC. Cells expressing CK8 (brown staining) were detected. Magnification 40 X. Second row: expression of CK18 (brown staining) was detected. A few cells expressed c-Met. hFLMSC did not express CK19 and Hep Ag. Magnification 40 X. (B) hFLMSC were characterized with regard to expression of CD44, CD 106, CD29, CD45, CD73, CD 166, CD54 and CD34 using flow cytometry. First row: hFLMSC showed strong expression of CD44 and weak expression of CD29. Expression of CD 106 and CD45 was not observed. Second row: expression of CD73, CD 166 and CD54 was strong on hFLMSC, while expression of CD34 was not detected.

hFLMSC from passages 6-11 (*n* = 5) were characterized by flow cytometry using monoclonal antibodies specific for several surface markers. hFLMSC were strongly positive for CD44. They were also positive for CD 166, CD73 and CD29 (Figure 2), but negative for ALB. Interestingly, hFH also showed high expression of CD44, CD 166, CD73 and moderate expression of CD29, but were, in contrast to the hFLMSC, strongly positive for ALB (data not shown). In addition, cultured hFH but not hFLMSC expressed CD34 (data not shown).

### hFLMSC do not express any hepatocyte markers

The expression of ALB, AFP, CK8, CK18, CK19 c-Met, and Hep Ag in cultured hFLMSC was examined using immunocytochemistry (Figure 2). MSC did not express ALB, CK19 and Hep Ag but were positive for CK8, CK18 and c-Met in culture.

### Levels of cytokine and chemokine levels in hFLMSC supernatants

**Figure 3:**
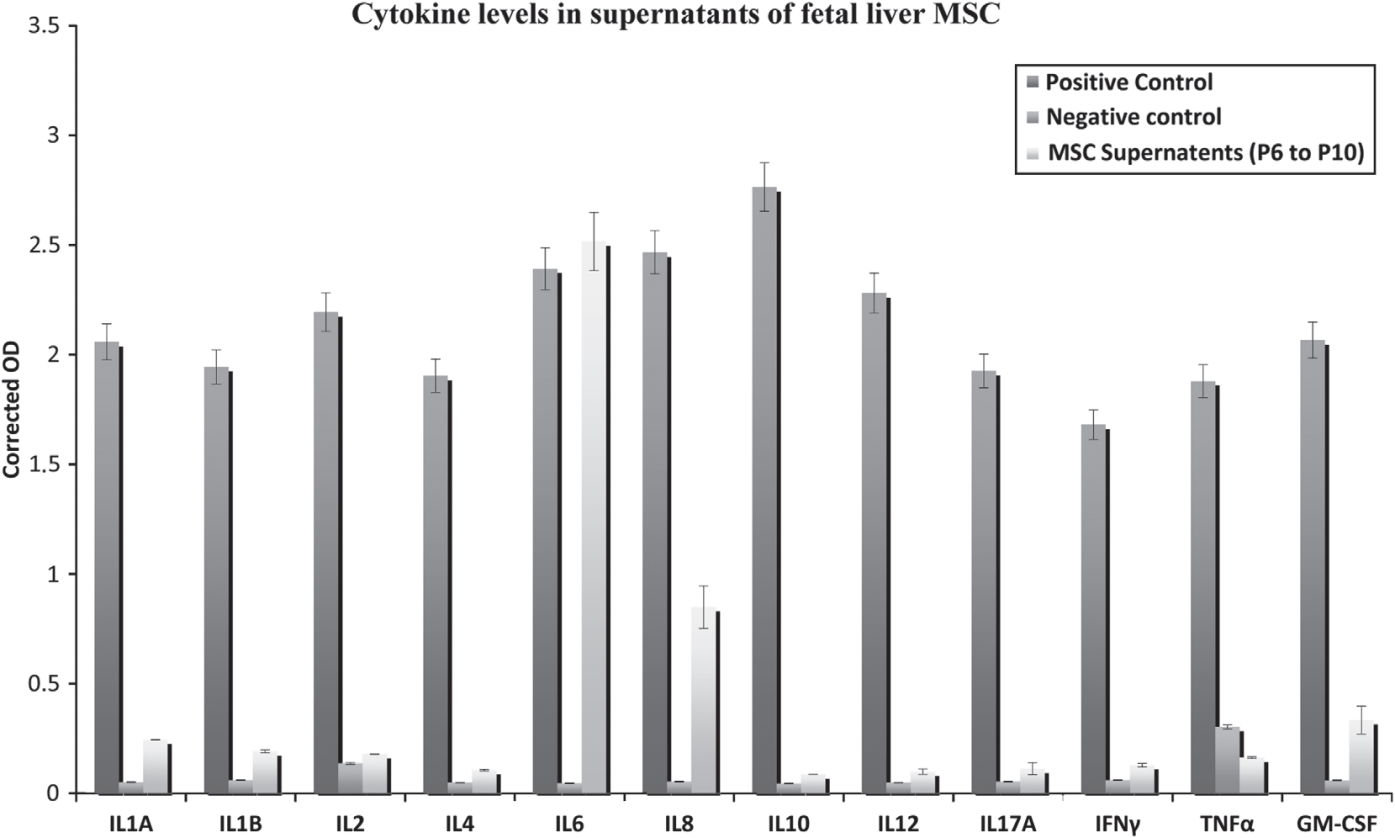
Detection of cytokines in hFLMSC culture supernatants. The levels of common cytokines in human hFLMSC culture supernatants were assessed using ELISA. Supernatants showed high levels of IL-6 and IL-8, while low levels of IL-1α, IL-1β and GM-CSF were detected.

The cytokines and chemokines analyzed by this array were IL-1α, IL-1β, IL-2, IL-4, IL-6, IL-8, IL-10, IL-12, IL-17A, IFN-γ, TNF-α and GM-CSF. We found that hFLMSC produced very high levels of the pro-inflammatory cytokine IL-6 in all cell passages tested. Besides IL-6, IL-8, GM-CSF and low levels of the IL-1 cytokines were detected in the supernatants of hFLMSC cultures (Figure 3).

### In vitro functionality of hFH and hFLMSC

Functionality of hFH and hFLMSC was assessed by measuring the ammonia and urea content and SOD activity (Table I) using adult hepatocytes as a positive control. In general, fetal cells produced extremely low amounts of these factors compared with adult hepatocytes. Nevertheless, a comparison between hFH and hFLMSC showed that the ammonia content was lower, while urea content was higher, in hFLMSC. Interestingly, hFH showed higher SOD activity than hFLMSC.

**Table I:** Cell lysates of hFH, mesenchymal cells and adult hepatocytes were analyzed with regard to ammonia and urea content, and SOD activity.

Groups	Ammonia (mg/mL)	Urea (mg/L)	SOD (U/mg protein)
Fetal hepatocytes	0.17±0.003^a^	0.10±0.02^a^	4.0±0.06^a^
GFP-expressing hFLMSC	0.16±0.002^a^	0.20±0.005^a^	0.2±0.03^a^
Adult hepatocytes	0.60±0.07	6.40±0.25	6.1±0.74

Results are mean ± SD.

a Compared with adult hepatocytes (*P* < 0.05), fetal hepatocytes from three different individuals; GFP-expressing MSC from three different passages; adult hepatocytes from one individual. Ammonia assay: two experiments done in duplicate and a second in triplicate. Urea assay: two experiments done in triplicate. SOD: one experiment done in quadruplicate.

### hFH and MSC engraft in retrorsine-injured mouse liver

**Figure 4:**
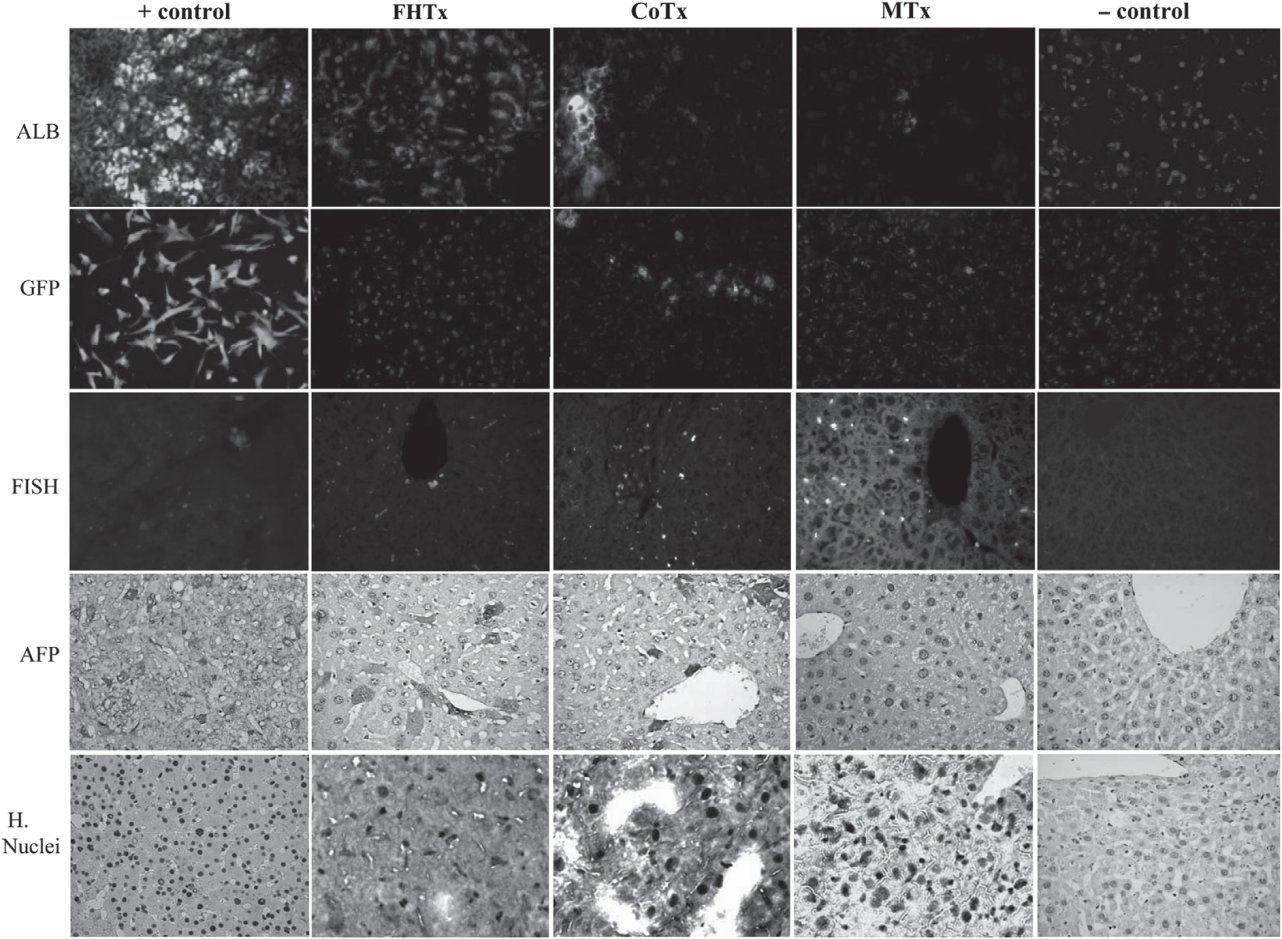
Transplantation of hFH and fetal liver MSC in nude mice. The FHTx group received 2 million hFH, the CoTx group received 1 million hFH and 1 million hFLMSC, and the MTx group received 2 million hFLMSC. Cells were transplanted into the spleen of retrorsine-treated nude mice that underwent 40% partial hepatectomy at the time of transplantation. Paraffin-embedded sections stained with human DNA-specific probe (FISH) showed the presence of human cells (red signals). Nuclei were counterstained with DAPI (blue). Immunohistochemistry was performed on freshly frozen liver sections from FHTx, CoTx and MTx groups, to detect human ALB staining (green staining). Paraffin-embedded sections were stained for human-specific AFP nuclear antigen. Biopsy of liver cancer served as a positive control and a sham group as a negative control. Hematoxylin (HE) counterstained. Magnification 40 X.

**Figure 5:**
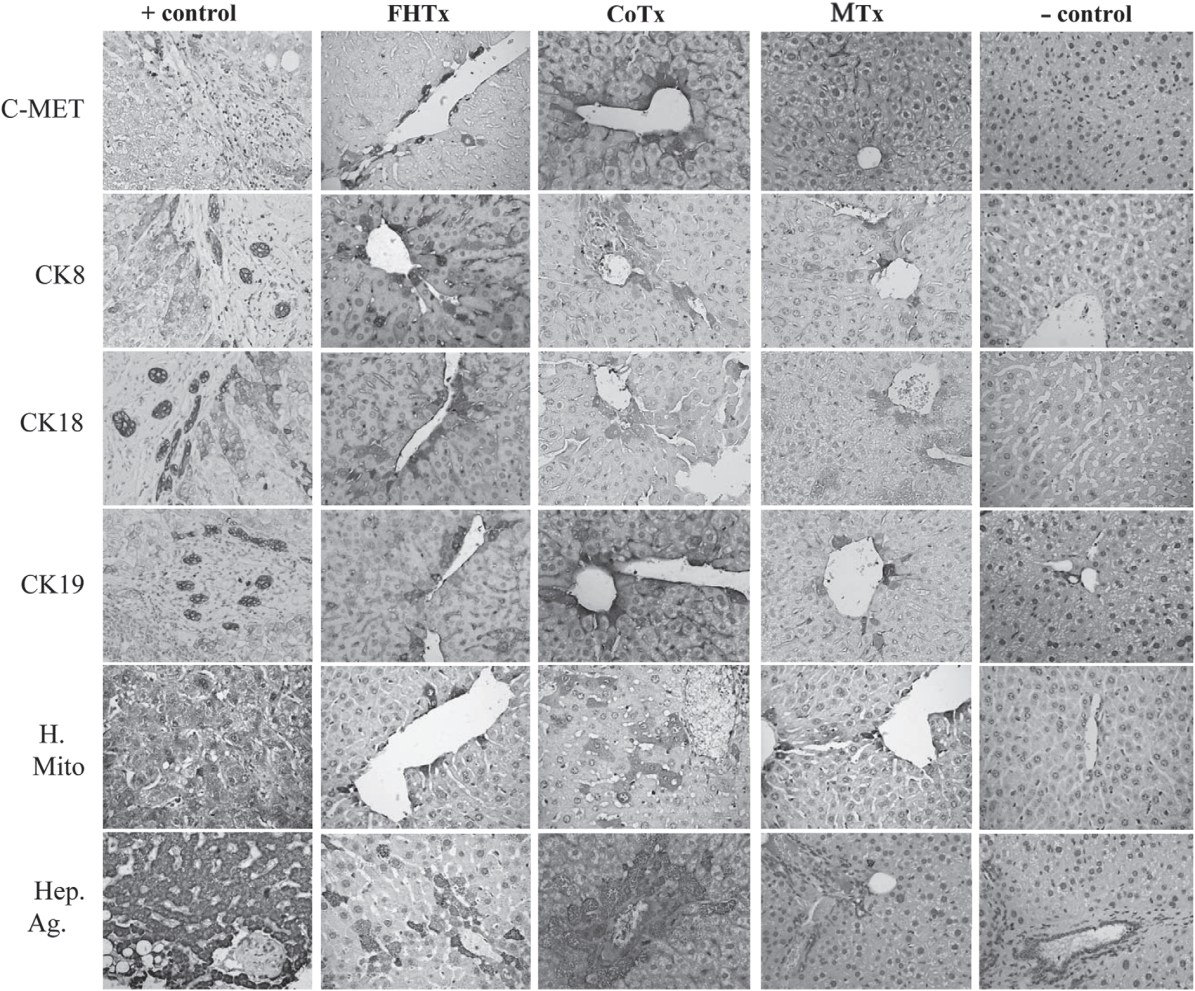
Transplantation of hFH and hFLMSC in nude mice. Paraffin-embedded sections were stained for human c-Met, CK8, CK18, CK19, mitochondrial antigen and hepatocyte antigen. Engrafted human cells stained dark brown. Biopsy sections from patients with liver cancer served as a positive control and a sham group as a negative control. HE counterstained. Magnification 40 X. Paraffin-embedded sections were stained for human-specific AFP nuclear antigen. Biopsy of liver cancer served as a positive control and a sham group as a negative control. HE counterstained. Magnification 40 X.

Engraftment of hFH and hFLMSC in mouse livers was verified by the use of antibodies specific for human ALB, AFP and nuclear antigen (Figure 4), and c-Met, CK8, CK18, CK19, mitochondrial antigen and Hep Ag (Figure 5). Scattered human ALB^+^ hepatocytes were detected between the periportal and centrilobular regions of livers in animals receiving only hFH (2 × 10^6^ hFH). Interestingly, liver sections from CoTx animals (1X10^6^ hFH+lX10^6^ hFLMSC) showed colonies, rather than scattered cells, of hepatocytes synthesizing ALB. Staining of liver sections from animals receiving only hFLMSC (2 × 10^6^ hFLMSC; MTx group) revealed extremely few cells positive for human ALB. FISH using a human-specific DNA probe was also used to detect human cells. The CoTx group had higher numbers of FISH + cells compared with groups receiving only hFH or hFLMSC (Figure 4). In contrast to the recipient livers of the mice in the hFH and CoTx groups, livers of mice in the MTx group did not show expression of c-Met or Hep Ag (Figure 5). The livers of the CoTx mice showed high numbers of cell colonies expressing human-specific antigen near the portal region (Figure 5).

### Detection of human-specific mRNA in recipient mouse livers using RT-PCR

**Figure 6:**
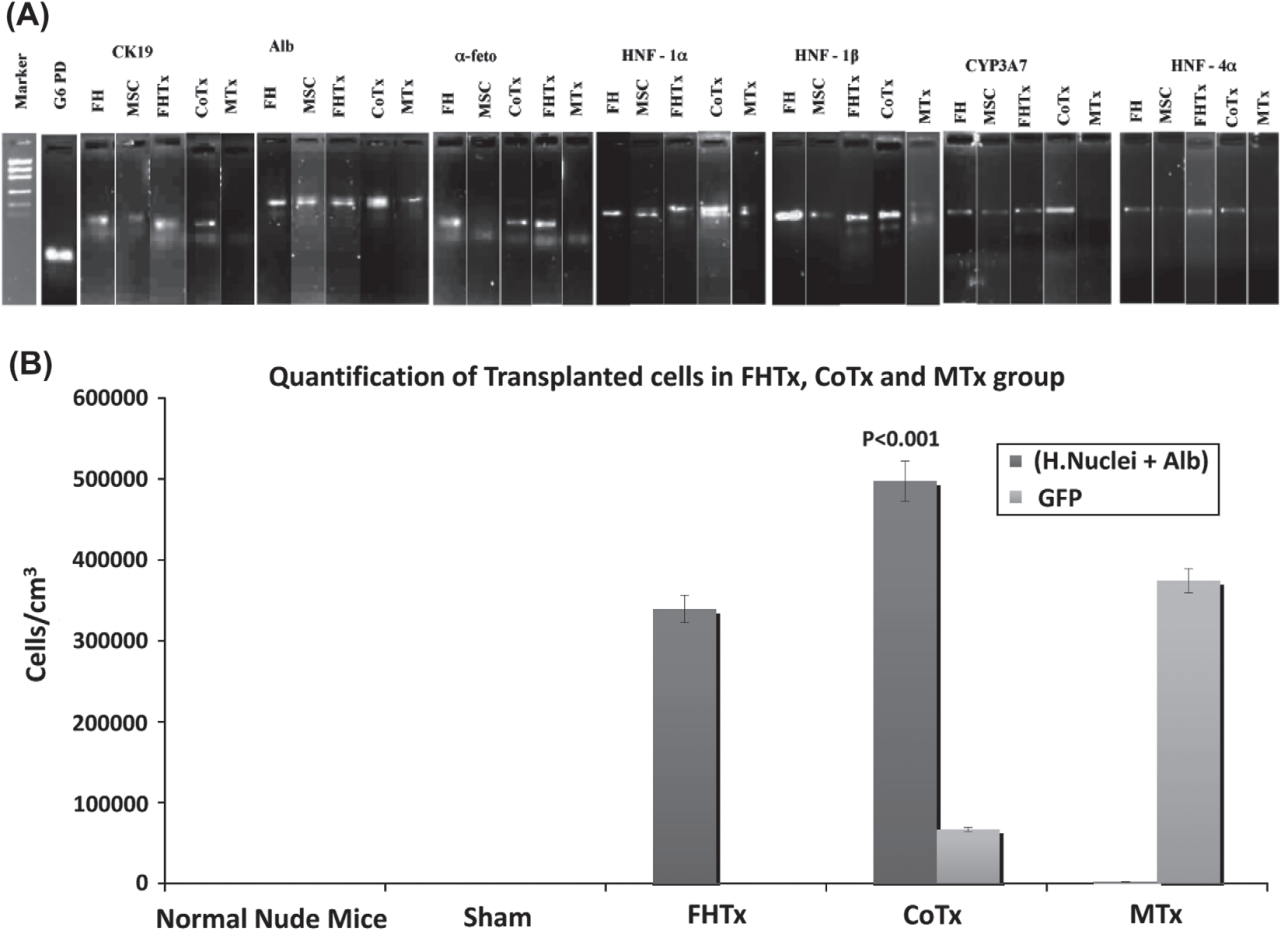
Human hepatocyte-specific gene expression as detected by RT-PCR and quantification of engrafted cells. (A) Expression of CK19, ALB, AFP, HNF-la, HNF-ip, HNF-4a and CYP3A7 mRNA was analyzed by RT-PCR of mouse livers after transplantation. The detoxifying enzyme CYP3A7 transcript and human-specific CK19, ALB, AFP, HNF-1 a, HNF-1P and HNF-4 a were specifically expressed in CoTx mouse livers. (B) Engrafted cells in the FHTx, CoTx and MTx groups were counted manually from five different liver regions and presented in cells in cm/mm^3^. Cells positive for human nuclei and ALB were highest in the CoTx group. GFP^+^ cells were highest in the MTx group.

RT-PCR was performed to detect the presence of human ALB, AFP, CK19, HNF-1α, HNF-1β, HNF-4α and CYP3A7 mRNA in recipient mouse livers of the hFH transplanted (FHTx), CoTx and MTx groups (Figure 6A). The CoTx group showed intense expression of ALB, HNF-1α, HNF-1β and CYP3A7, and moderate expression of CK19, AFP and HNF-4α. Interestingly, the MTx group did not show detectable expression of HNF-4α, CYP3A7, AFP or CK19. Moderate expression of all genes was observed in the FHTx group.

### Human ALB concentration in transplanted mouse sera

The human ALB concentration in the serum of transplanted mice was highest (3.15 ± 0.008 ng/mL) in the CoTx group, and significantly (*P*< 0.01) lower in the FHTx group (2.50 ± 0.008 ng/mL) and not detected in the MTx group (Table II).

**Table II:** Human ALB and soluble human c-Met concentrations in various groups.

Groups	Human ALB (ng/mL)	Soluble human c-Met (ng/mL)
Normal nude mice	ND	ND
Sham	ND	ND
Transplantation (Tx)		
FHTx	2.50±0.008^a^	0.65±0.003
CoTx	3.15±0.008^b^	0.96±0.002^c^
MTx	ND	0.50±0.006

Results are mean ± SD (*n* = 5). ND, not detectable.

a Compared with CoTx (*P* < 0.05).

b Compared with MTx and FHTx (*P* < 0.01).

c Compared with FHTx and MTx (*P* < 0.001).

### Human c-Met in liver homogenates of transplanted mice

We found that the c-Met concentration was higher in mouse livers of the CoTx group (0.96 ±0.002 ng/mL) compared with the FHTx (0.65 ±0.003 ng/mL; p<0.05) and MTx (0.50 ±0.006 ng/mL; *P<* 0.01) groups. Lysates from normal and cancerous human liver tissue were used as controls (Table II).

### Quantification of engrafted hFH and hFLMSC

We found that the magnitude of liver repopula-tion with regard to human cells carrying hepa-tocyte markers was highest in the livers of mice in the CoTx group. The number of double-positive cells [human (H.) nuclei^+^ ALB^+^] in this group was 81 000±2103/mm^3^ compared with 58 300 ± 8800/mm^3^ *(P<* 0.001) in the FHTx group and 1329 ± 156/mm^3^ in the MTx group. In the MTx group more single-positive (H.nuclei^+^ ALB") cells were found (Figure 6B and Table III). As expected, higher numbers of GFP^+^ cells were found in the MTx group (71 400 ± 2000) compared with the CoTx group (23 000 ± 1000). Interestingly, Ki67 and CK8 double-positive cells were few in the livers of mice in the FHTx and CoTx groups, suggesting that transplanted cells did not proliferate to a large extent. In particular, the MTx group had very few proliferating cells (22 ±13). Liver sections from sham transplanted and normal mice did not show the presence of any human cells (Table III).

**Table III:** Quantification of engrafted human cells in livers of nude mice with acute liver injury.

	Cells/mm^3^			
Groups	H.nuclei^+^ ALB^+^	H.nuclei^+^ ALB^−^	GFP^+^	Ki67^+^ CK8^+^
Normal nude mice	ND	ND	ND	ND
Sham Tx	ND	ND	ND	ND
FHTx	58 300±8800	1772±195	ND	66 ± 15
CoTx	81 000±2103^a^	16 000±300	23 000±1000	155 ± 32^a^
MTx	1329±156	47 600±5500	71 400±2000^b^	22 ± 13

Results are mean ± SD (*n* = 4). ND, not detectable.

a Compared with FHTx and MTx (*P* < 0.001).

b Compared with CoTx and FHTx (*P* < 0.01).

## Discussion

In clinical hepatocyte cell transplantation, engraft-ment of these cells in the diseased liver is influenced by many factors, including deficiency of nutrients, hypoxia, oxidative stress, inflammatory response and fibrosis. The environment is harsh in the damaged liver, where vascular structures are damaged and trophic support is lacking. Death of the transplanted hepatocytes further intensifies the harshness of the microenvironment by priming immune and inflammatory responses. It is therefore important to optimize the cell transplantation conditions to withstand the severe conditions, especially during the acute phase after cell infusion. Thus successful hepatocyte cell therapy will depend on (a) use of highly proliferative cells and (b) engineering a situation by which the transplanted cells have a selective expansion advantage when introduced into the ischemic environment, to allow a time window long enough for the cells to acclimatize and expand. In the present study, we used a novel approach where we combined both these factors to achieve improved expansion of the transplanted hepatocytes. We co-transplanted highly proliferative hFH together with ‘facilitator cells’, namely human fetal liver-derived MSC.This cellular strategy resulted in higher engraftment compared with either of the populations transplanted alone. The CoTx group showed a 60-fold increased hepatocyte expansion and the total cells engrafted per mm^3^ was 81 000 ±2103 (calculated as double-positive cells in CoTx/ double-positive cells in MTx), which equals 12% engraftment of the injected cells. On the other hand, in the FHTx group we found that 6% of the transplanted cells engrafted, while in the MTx group 7% engrafted. These findings support our hypothesis that hFLMSC facilitates hFH engraftment.

To test our hypothesis that the hFLMSC may act as facilitator cells that augment hFH engraftment after transplantation, we carried out the following. We first injured the native liver with an injection of retrorsine, followed by 30–40% partial hepatectomy (see the Methods). Previously (6), we have routinely used a 70% Partial Hepatactomy (PH) model. This approach results in the availability of an extremely small liver mass for engraftment of newly injected cells. The purpose of performing PH is to release important growth factors required for successful regeneration of the injured liver (15, 16). Therefore, in the present study we performed a 30% PH in order to increase the total volume of the native liver mass and, together with the released growth factors, facilitate the successful engraftment of the injected cells. We infused 2X 10^6^ (representing 0.5% of the host liver mass) hFH and/or MSC into the spleen to facilitate blood flow-mediated translocation to the hepatic mass. Immediately after injection, we observed transient hypertension in all animals as a result of entrapped cells. However, entrapped cells have been shown to be cleared spontaneously within 24 h (17) with no subsequent ill effects for the animal. All the above-mentioned modifications helped improve our overall animal survival rates. We confirmed the presence of engraftment and repopulation with human cells in the experimental model by immu-nohistochemistry and PCR. Immunohistochemical analysis showed that the co-transplanted animals had a higher number of cell colonies positive for human ALB, AFP, c-Met, hepatocyte antigen, CK8, CK18 and CK19. We also detected higher levels of human serum ALB in the CoTx group. Furthermore, RT-PCR analysis showed intense expression of the important hepatic factors HNF-1α,β, HNF-4α, and CYP3A7 and human ALB in the livers of CoTx compared with only hFH and MSC. The expression of transcription factors in the liver of CoTx animals provides useful information regarding the persistence of hepatocyte function and differentiation (18).

Prior to injection of the two cell types, we characterized both populations. Our, immunocy-tochemical analysis showed expression of several liver-specific markers in hFH; however, hFLMSC did not show expression of ALB, CK19 and Hep Ag, although they did express c-Met, CK8 and CK18. *In vitro* measurement of hFH and hFLMSC cell cultures for ammonia, urea content and SOD activity demonstrated that, in general, these cells had significantly lower values compared with adult hepatocytes, indicating the immature nature of the fetal cells. This is not surprising because in fetal life energy requirements are fulfilled via maternal blood; at birth this supply is interrupted and replaced by an external supply, thus the detoxification system evolves after birth (19).

An important observation is that in our study an extremely low number of MSC demonstrated hepatic markers, indicating that MSC do not have the capacity to transdifferentiate into hepatocytes, as has been suggested by other studies (20). We would like to argue that if fetal liver MSC under appropriate conditions do not differentiate into hepatocytes, then the probability of transdifferentiation of MSC from other tissues into hepatocytes is even lower. However, our results demonstrate that MSC facilitate the expansion of hepatic progenitor cells. An important issue that needs to be addressed is the mechanism by which these cells augment hepatocyte expansion. Our preliminary data demonstrate that screening of hFLMSC supernatants at various passages for different chemokines and cytokines showed significantly high levels of IL-6 and IL-8, but low levels of TNF-α and IL-1. It has been reported that MSC secrete high levels of IL-6 as an anti-inflammatory cytokine, which is mediated through inhibitory effects onTNF-a and IL-1 (21, 22). Thus the high levels of IL-6 and IL-8 produced by MSC may counterbalance the hepatocyte transplantation-induced liver inflammation caused by pro-inflammatory cytokines produced by Kupffer cells and infiltrating neutrophils (23). Furthermore, metalloproteinases produced by MSC may induce vascular permeability allowing larger numbers of hepatocytes to translocate quickly through the sinusoids and integrate efficiently into the liver parenchyma (24, 25). It is important to state that, in our experience, MSC derived from bone marrow do not significantly improve expansion compared with fetal liver MSC (Sumitran-Holgersson, unpublished data). This finding reflects that tissue-specific factors produced by MSC may play an important role in efficient engraftment. It is probable that for future successful clinical hepatocyte transplantation, liver-derived MSC may be the best candidates for improving engraftment of the infused hepatocytes. We are currently elucidating the mechanisms by which fetal liver-derived MSC augment hepatocyte expansion.

In summary, our study reports the development of a novel strategy for augmenting hepatocyte cell engraftment by co-transplanting liver-derived MSC with hepatocytes. This approach is promising and thereby advances this experimental procedure closer to clinical liver cell therapy.

## Supplementary material available online

Supplementary Table I to be found online at http://www.informahealthcare.com/doi/abs/10.3109/14653249.2012.663526
