# Supplementary material for: Fetal liver-derived mesenchymal stromal cells augment engraftment of transplanted hepatocytes
Source: Cytotherapy. 2012 Mar 16;14(6):657–69. doi: 10.3109/14653249.2012.663526 (PMC3411318; doi:10.3109/14653249.2012.663526)
Supplement: Supplementary file 1 [file mcyt14-657-SD1.pdf]

Supplementary Table 1. Primer sequences used for detection of mRNAs for hepatocyte-specific markers together with the expected product size, the annealing temperature and number of cycles used.

Transcript	Primer sequence	Product size	Annealing temperature	Cycles
Human ALB	TGC TTG AAT GTG CTG ATG ACA GGG<br>AAG GCA AGT CAG CAG GCA TCT CAT	161 bp	58° C	38
CYP3A4	CAA GAC CCC TTT GTG GAA AA<br>CGA GGC GAC TTT CTT TCA TC	187 bp	55° C	40
CYP3A7	AAG GTC GCC TCA AAG AGA CA<br>TGC ACT TTC TGC TGG ACA TC	223 bp	53° C	33
HNF-4α	CCA AGT ACA TCC CAG CTT TC<br>TTG GCA TCT GGG TCA AAG	295	55° C	40
HNF-1α	CCA TCC TCA AAG AGC TGG AG<br>GTT GAG GTG TTG GGA CAG GT	197	53° C	35
HNF-1β	CAA CCA GAC TCA CAG CCT GA<br>TGC CAT GGT GAC TGA TTG TT	152	53° C	35
CK-19	CCTGCGGGACAAGATTCTTG<br>ACGGGCGTTGTCGATCTG	70	53° C	35
AFP	GCAAAGCTGAAAATGCAGTTGA<br>GGAAAGTTCGGGTCCCAAAA	129	53° C	35
G6PD	TGC CCC CGA CCG TCT AC<br>ATG CGG TTC CAG CCT ATC TG	76	53° C	35

ALB, albumin; CYP, cytochrome P450; HNF, hepatocyte nuclear factor; CK, cytokeratin; AFP, alpha-fetoprotein
